# Inpatient Management of a Monoamniotic Twin Pregnancy Complicated by Umbilical Cord Entanglement and Selective Intrauterine Growth Restriction

**DOI:** 10.7759/cureus.31215

**Published:** 2022-11-07

**Authors:** Noah F Gomez, Hope Woodroffe

**Affiliations:** 1 Obstetrics and Gynecology, Sisters of Charity Hospital, Buffalo, USA

**Keywords:** high risk pregnancy, fetal surveillance, intrauterine growth restriction, umbilical cord entanglement, monoamniotic twins

## Abstract

Monoamniotic twin pregnancies are exceedingly rare. These are high-risk pregnancies that present a unique set of challenges for the obstetrician-gynecologist. There is a high risk of intrauterine fetal demise, secondary to co-morbid conditions including, but not limited to, congenital anomalies and umbilical cord entanglement. Successful delivery and favorable neonatal outcomes are predicated on early diagnosis, intensive fetal monitoring, and timely delivery. The management guidelines for these pregnancies rely primarily on retrospective studies and expert consensus, although some substantial conclusions can be made regarding appropriate antepartum and intrapartum care. We present the case of a 38-year-old gravida four, para three, with monoamniotic twins who delivered successfully after inpatient hospitalization at viability, administration of corticosteroids for fetal lung maturity, and vigilant fetal monitoring. We conclude that the combination of early ultrasound, intensive fetal monitoring, and interdisciplinary coordination among generalist obstetrician-gynecologists, maternal-fetal medicine specialists, and nursing staff is paramount for providing the greatest chance of a favorable outcome.

## Introduction

Monoamniotic twins are a rare occurrence, with an incidence of about one in 10,000 pregnancies [1]. Monoamniotic pregnancies result from the division of the embryonic disc between days nine and 13 post-fertilization, therefore both fetuses share the same placenta and amniotic sac [2]. These high-risk pregnancies may be complicated by a variety of conditions, including twin-twin transfusion syndrome (TTTS), twin reversed arterial perfusion sequence, intrauterine growth restriction (IUGR), congenital malformations, and exclusive to monoamniotic pregnancies, umbilical cord entanglement [1]. The mortality rate is as high as 30% [2]. Furthermore, the management of monoamniotic gestations relies heavily on retrospective studies and expert opinion, secondary to its rarity. Here we present the case of a monoamniotic twin pregnancy where two live babies via repeat cesarean section at 32 weeks were delivered.

## Case presentation

A 38-year-old gravida four, para 3, presented to our office at approximately 10 weeks and three days gestation by last menstrual period for initial prenatal visit. Bedside ultrasound was significant for a live intrauterine twin gestation with no clear dividing membrane. The patient had a past history of three previous low transverse cesarean sections. The indications of the first two C-sections were fetal distress and cephalopelvic disproportion, respectively. The indication of the third cesarean was a history of two previous C-sections. Her three previous pregnancies were otherwise uncomplicated. The patient denied a history of abnormal pap smears, or sexually transmitted diseases, including gonorrhea, chlamydia, and herpes. The patient denied any significant past medical history, tobacco, drug, or alcohol use. Previous surgeries included wisdom tooth extraction and three previous C-sections. Current medications included a prenatal vitamin and stool softener, and the patient had no known drug allergies. Prenatal labs were unremarkable. This pregnancy was also complicated by advanced maternal age, although the patient declined genetic testing.

**Figure 1:**
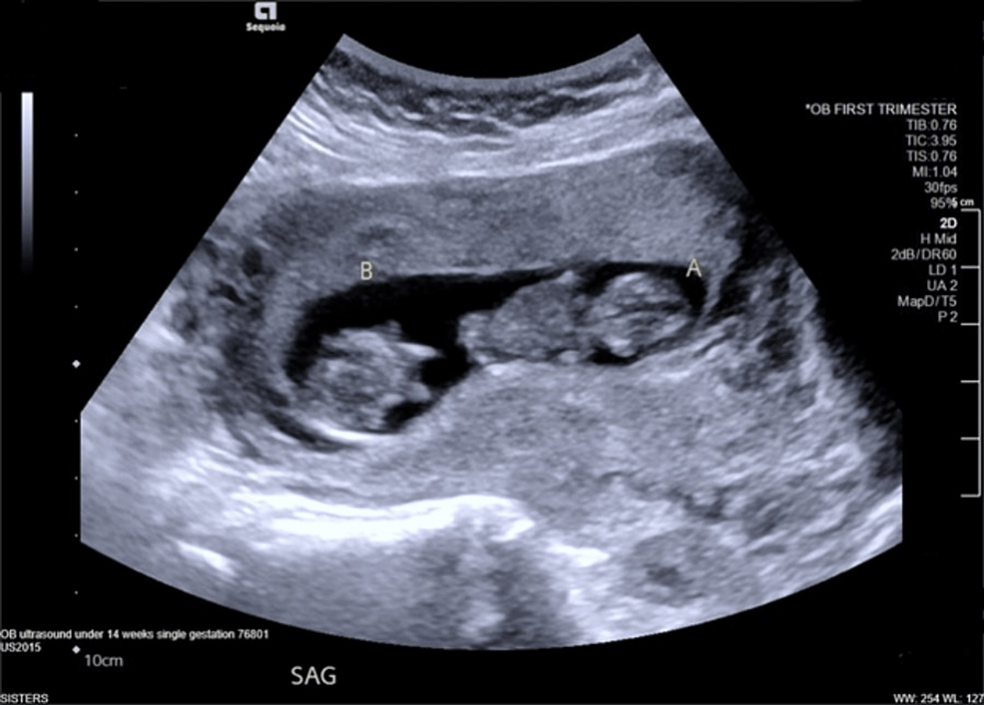
Dating ultrasound at 11 weeks showing twin intrauterine gestation without an apparent dividing membrane

The official first-trimester ultrasound did confirm a monoamniotic pregnancy (Figure 1). Repeat ultrasound was performed every 2-4 weeks. Mild cord entanglement was first noted on 17-week ultrasound. The anatomy scan for both fetuses appeared normal. At 23-week gestation, fetus B demonstrated IUGR with an estimated fetal weight (EFW) of 464g (1lb 0oz), in the 9th percentile, with normal umbilical artery (UA) and middle cerebral artery (MCA) dopplers. 

The patient was admitted to the antepartum unit at 24 weeks with hospitalization planned until repeat C-section, which was anticipated between 32-34 weeks. At the time of admission, she had good fetal movement and denied any vaginal bleeding, discharge, leakage of fluid, cramping, or contractions. Upon admission, the patient received betamethasone for fetal lung maturity. Initial fetal surveillance included daily biophysical profiles (BPPs), non-stress tests (NSTs) three times daily, weekly UA and MCA dopplers, and biweekly fetal weights. Furthermore, the patient was started on subcutaneous enoxaparin 40mg daily for deep vein thrombosis prophylaxis and a peripherally inserted central catheter was placed. Her hospital course was complicated by iron deficiency anemia, with a hemoglobin of 10.4 on admission, which resolved status-post oral iron supplementation and 200mg IV iron sucrose infusions for two days. 

**Figure 2:**
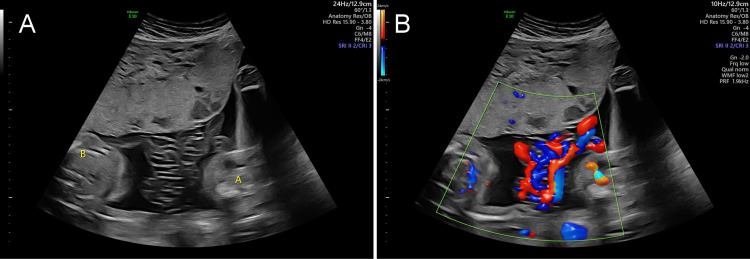
Early second-trimester ultrasound demonstrating significant cord entanglement, without color doppler in (A) and with color doppler in (B)

Ultrasound at 25 weeks was significant for a fetal weight discordance of 20%, with fetus B still growth restricted at the 9th percentile. UA and MCA dopplers were normal for both fetuses. The cord entanglement was stable and did not appear to include fetal appendages or the fetuses themselves (Figure 2). Follow-up ultrasound two weeks later showed resolution of growth restriction in fetus B, with an EFW in the 46th percentile.

At 27 weeks and four days, the patient had an episode of irregular contractions every 10-15 minutes, associated with intermittent variable decelerations in fetus A. The patient was then monitored continuously for the next few hours until contractions and decelerations resolved, status post IV fluid bolus. A urine culture was obtained, which resulted positive for *Proteus mirabilis* two days later. The patient was started on daily antibiotic treatment. Roughly 24 hours later, the patient began to experience regular contractions every 5-10 minutes, without bleeding or leakage of fluid. Contractions were refractory to IV fluid bolus and the patient was found to be 4cm dilated, 50% effaced, -3 station, without any previous cervical exams for comparison. The patient was transferred to the labor and delivery unit for continuous fetal monitoring, rescue betamethasone, and magnesium for neuroprotection. The contractions eventually self-resolved without any change in the cervical exam, and the status of the fetuses remained reassuring; therefore, the patient was transferred back to the antepartum unit. After five days of treatment with IV Rocephin 1g daily, repeat urine culture was negative. 

Ultrasound at 29 weeks showed severe IUGR less than the 3rd percentile in fetus A, with UA dopplers higher than MCA dopplers, consistent with a head-sparing pattern; therefore, the frequency of fetal dopplers was increased to twice weekly, with planned delivery now between 32 and 33 weeks. Final growth ultrasound at 31 weeks showed fetus A with an EFW in the 7th percentile and fetus B with an EFW in the 19th percentile, with normal dopplers for both fetuses. Both fetuses were in the cephalic position from 27 weeks until delivery.

Throughout the patient’s admission, BPPs were consistently reassuring, and NSTs were mostly reactive without any decelerations, although there were a few occasions of intermittent variable decelerations that resolved spontaneously or with IV fluid boluses. The amniotic fluid level remained within normal limits, and the cord entanglement was stable. The placenta was anterior and distant from the lower uterine segment without evidence of placenta accreta spectrum. The fetuses did not demonstrate evidence of TTTS.

**Figure 3:**
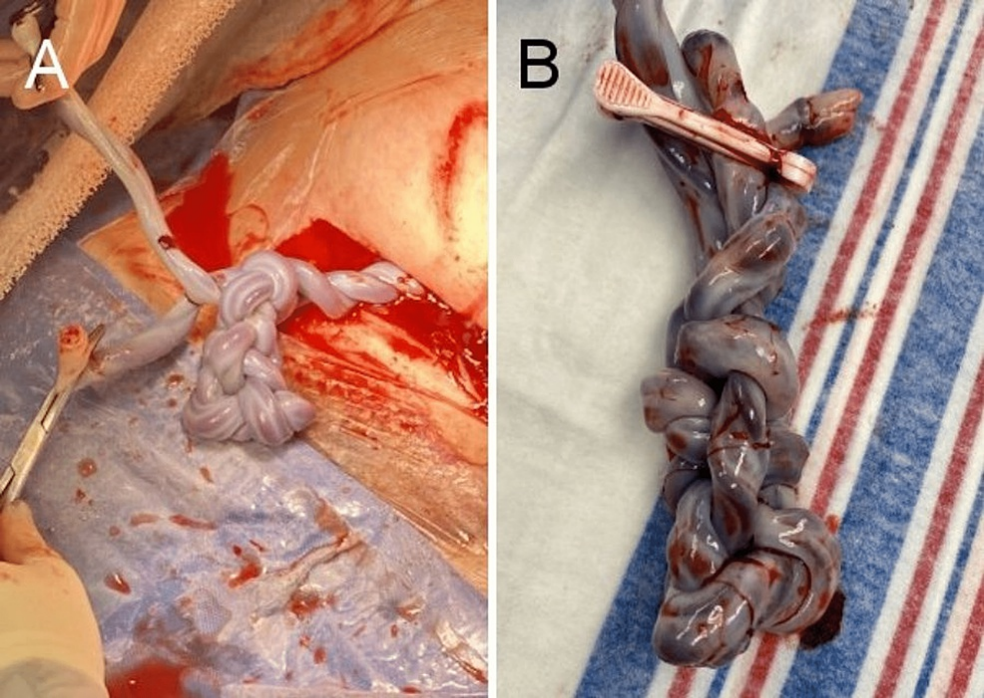
Entangled umbilical cords immediately after delivery of both newborns (A) demonstrating a complex knotted pattern with multiple intertwined loops, also visible in a magnified view (B) after delivery of the placenta

**Figure 4:**
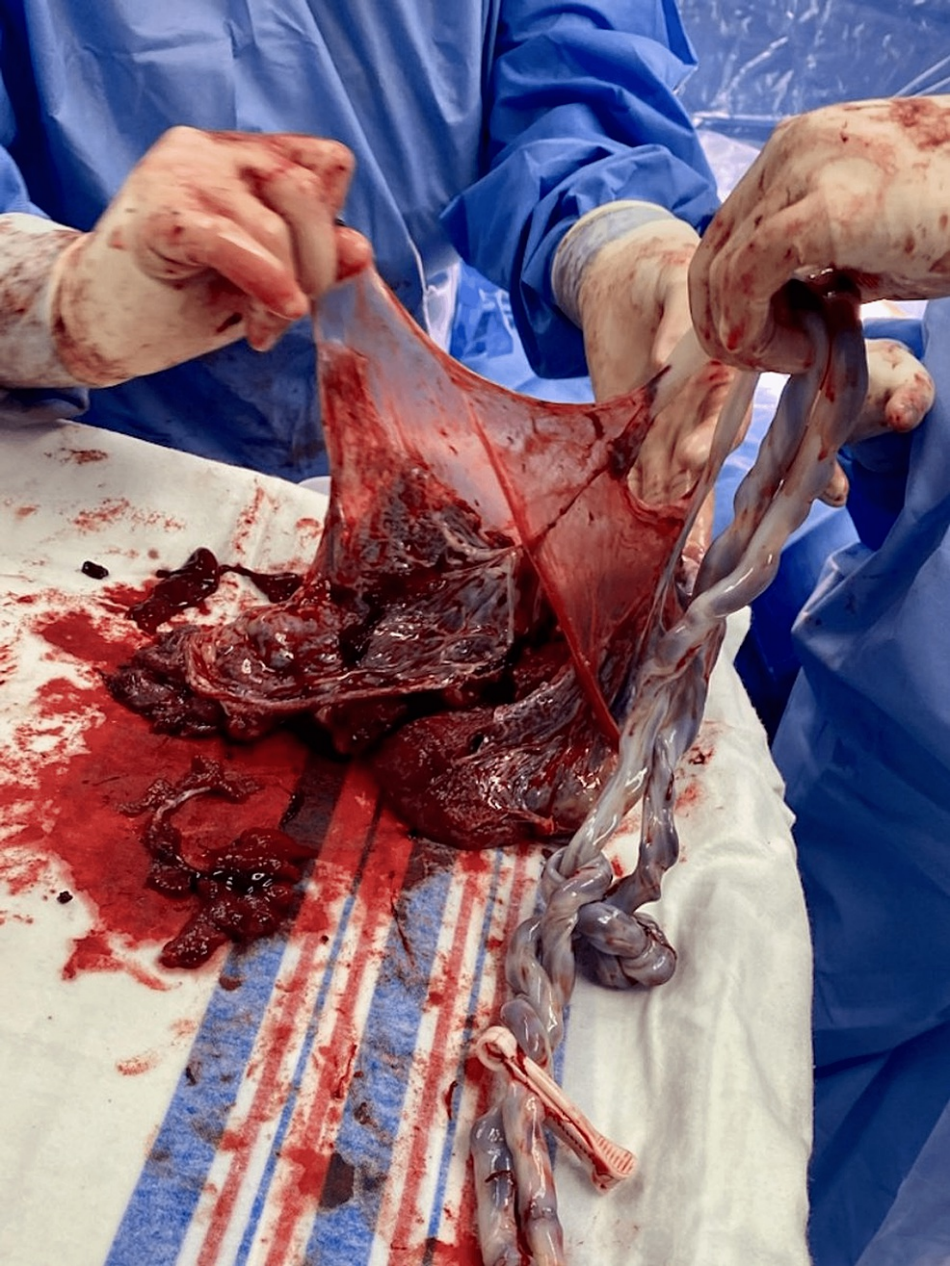
Placenta ex-utero, with a single amniotic membrane

Repeat C-section was performed at 32 weeks in light of cord entanglement, selective IUGR, and advanced cervical dilation. Surgery was uncomplicated and blood loss was 845mL. Baby A delivered in the cephalic position, weighing 1520g (3 lb 5.6 oz) with an Appearance, Pulse, Grimace, Activity, and Respiration (APGAR) score of five at one minute and eight at five minutes. Cord gas pH was 7.34 with a base excess of 0.0. Baby B was delivered in the cephalic position, weighing 1675g (3 lb 11.1 oz) with an APGAR score of five at one minute and seven at five minutes. Cord gas pH was 7.35 with a base excess of 0.3. The amniotic fluid was clear for both babies. Both umbilical cords contained three vessels. The umbilical cords appeared entangled in a complex knot with multiple intertwining loops (Figure 3). The placenta was delivered in pieces and approximated ex-utero, and appeared normal (Figure 4).

The uterus, fallopian tubes, and ovaries appeared normal. The low transverse uterine incision was closed in two layers. Final pathology showed one placental disk and one amniotic sac. The cord of baby A had a furcate marginal insertion and measured 51cm long. The cord of baby B was 45cm long and was inserted on the placenta 4 cm away from the cord of baby A, with a paramarginal insertion 1 cm from the nearest disc edge. Fetal membranes showed no pathological change. Both babies were transferred to the neonatal ICU (NICU) in stable condition. The patient’s postoperative course was uncomplicated and she was discharged from the hospital on postoperative day two. 

## Discussion

The appropriate management of monoamniotic twins is still unclear and varies among clinicians [3]. There appears to be agreement that prior to viability, early confirmation of chorionicity and amnionicity, as well as identification of fetal anomalies should be performed [4]. After viability, there is consensus that corticosteroids should be administered for fetal lung maturity, and both the American College of Obstetricians and Gynecologists (ACOG) and the Royal College of Obstetricians and Gynecologists (RCOG) agree that delivery should occur between 32-34 weeks gestation, around which time the risk of intrauterine fetal demise is thought to outweigh the risk of prematurity [5,6,7]. It appears that hospital admission and concomitant intensive fetal monitoring (nonstress test [NST] at least three times daily) have improved outcomes compared to outpatient management [8]. In fact, as of 2015, there were only three recorded intrauterine fetal demises in monoamniotic twin pregnancies managed in an inpatient setting, compared to a rate of about 8% for those managed outpatient [8]. Therefore, we recommended hospitalization to our patient due to the improved survivability, ability to quickly escalate fetal surveillance if needed, and capability to perform emergent surgery for fetal distress.

Theoretically, continuous fetal monitoring would identify any acute changes, necessitating delivery, although this has been shown to be impractical, with both fetuses being monitored only 51% of the time in one study [9]. Furthermore, studies have suggested that worsening of umbilical cord entanglement may be a subacute event identifiable with intermittent fetal monitoring, therefore obviating the ostensible benefits of continuous monitoring [10]. Presumably, changes in the fetal heart rate tracing indicative of cord compression include recurrent spontaneous variable decelerations or bradycardia, which would prompt expeditious delivery [9]. There have been studies that propose the use of the nonsteroidal anti-inflammatory drug sulindac as a means of medical amnioreduction, in the hopes of stabilizing fetal lie to prevent a cord accident [11,12]. Although promising, this should be considered experimental therapy.

While hospital admission may impose a non-negligible psychological burden on the mother [13], our case highlights benefits to the pregnancy afforded with inpatient care, namely the management of the patient’s episode of preterm contractions, which necessitated prompt transfer to the labor and delivery unit and administration of magnesium and rescue steroids, with concomitant treatment of the inciting urinary tract infection.

## Conclusions

Monoamniotic twins are an extremely rare and unique occurrence, which presents a complex challenge for physicians. Successful management requires close coordination with maternal-fetal medicine specialists and increased care from nursing staff. While not all fetal deaths may be preventable, intuitive steps such as early ultrasound and intensive fetal monitoring provide the greatest hedge against unpredictable complications. The most feared complication, umbilical cord entanglement, appears to be inevitable, although not as ominous as once thought. Unfortunately, the optimal fetal surveillance protocol to avert adverse antenatal events may never be based on prospective studies due to the strikingly low incidence of monoamniotic twins. Therefore, continued reporting of monoamniotic pregnancies in conjunction with research into the pathophysiology of cord entanglement is necessary to elucidate novel treatments and antenatal surveillance protocols.
